# Effect of the *Salmonella* Pathogenicity Island 2 Type III Secretion System on *Salmonella* Survival in Activated Chicken Macrophage-Like HD11 Cells

**DOI:** 10.1371/journal.pone.0029787

**Published:** 2011-12-27

**Authors:** Amanda L. S. Wisner, Andrew A. Potter, Wolfgang Köster

**Affiliations:** 1 Vaccine and Infectious Disease Organization, University of Saskatchewan, Saskatoon, Canada; 2 Canadian Center for Vaccinology, Izaak Walton Killam Health Centre, Halifax, Canada; Universite de la Mediterranee, France

## Abstract

In order to better identify the role of the *Salmonella* pathogenicity island 2 (SPI-2) type III secretion system (T3SS) in chickens, we used the well-known gentamicin protection assay with activated HD11 cells. HD11 cells are a macrophage-like chicken cell line that can be stimulated with phorbol 12-myristate 13-acetate (PMA) to exhibit more macrophage-like morphology and greater production of reactive oxygen species (ROS). Activated HD11 cells were infected with a wild-type *Salmonella enterica* subspecies *enterica* serovar Typhimurium (*S.* Typhimurium) strain, a SPI-2 mutant *S.* Typhimurium strain, a wild-type *Salmonella enterica* subspecies *enterica* serovar Enteritidis (*S*. Enteritidis) strain, a SPI-2 mutant *S.* Enteritidis strain, or a non-pathogenic *Escherichia coli* (*E. coli*) strain. SPI-2 mutant strains were found to survive as well as their parent strain at all time points post-uptake (PU) by the HD11 cells, up to 24 h PU, while the *E. coli* strain was no longer recoverable by 3 h PU. We can conclude from these observations that the SPI-2 T3SS of *S.* Typhimurium and *S.* Enteritidis is not important for survival of *Salmonella* in the activated macrophage-like HD11 cell line, and that *Salmonella* must employ other mechanisms for survival in this environment, as *E. coli* is effectively eliminated.

## Introduction

Infections by *Salmonella enterica* subspecies *enterica* are one of the leading causes of food borne gastroenteritis in humans [1]. Among those serovars responsible for food poisoning in humans, serovars Typhimurium (*S.* Typhimurium) and Enteritidis (*S.* Enteritidis) are most commonly isolated serovars from both humans and animals in many regions. In North America, *S.* Typhimurium is the primary serovar isolated from both humans and animals, while *S.* Enteritidis is the second most common serovar isolated from humans. The opposite is true for most of the European Union, with *S.* Enteritidis being the number one isolate from both humans and animals and *S.* Typhimurium being number two [2]. Both *S.* Typhimurium and *S.* Enteritidis are capable of causing systemic disease in humans, although this is not the normal course of infection and only occurs in very young, very old, and immunocompromised individuals [3].

*Salmonella* uses two specialized type III secretion systems (T3SS) that facilitate invasion and survival within the host cell. These two T3SSs are encoded within *Salmonella* pathogenicity islands 1 and 2 (SPI-1 and SPI-2) and secrete effectors into the host cell, triggering a number of events in the infected cell. These events ultimately lead to the symptoms of disease. It is the current view that the SPI-1 T3SS is mainly involved in invasion of the host cell, while the SPI-2 T3SS plays a role in survival within the host cell and maintenance of the *Salmonella* containing vacuole (SCV) [4], [5], [6]. SPI-2 is a region of approximately 40 kb and has been reported to be necessary for systemic infection, intracellular proliferation and survival, and maintenance of the SCV. However, the majority of these studies have been performed in mice, where *S.* Typhimurium and *S.* Enteritidis produce a typhoid-like infection rather than gastroenteritis, and therefore may not be indicative of the normal course of infection in healthy adult humans and chickens [7], [8], [9], [10].

The preferred site of invasion for *Salmonella* is through microfold (M) cells of the intestine. M cells reside within the follicular associated epithelium that overlays the Peyer's patches, have a less pronounced brush boarder, and are associated with mucous in less abundance than other intestinal epithelial cells. Once through the epithelial barrier, *Salmonella* are taken up by resident or recruited macrophages and dendritic cells [11], [12], [13], [14], [15]. An effective innate immune response is necessary to clear *Salmonella* and prevent systemic spread; recruited macrophages, natural killer cells and dendritic cells are paramount in this process, but in some cases *Salmonella* is able to manipulate and evade the host immune response and spread systemically [3], [12], [16], [17]. Within phagocytic cells, SPI-2 effectors are secreted across the SCV membrane and stop the fusion of lysosomes with the SCV, thereby avoiding bacterial killing by defensins, cathelicidins, lysozymes, lipases, proteases [18], [19], [20], [21]. This action is also thought to prevent the ability of reactive oxygen and nitrogen species (ROS and RNS) to form [18], [21]. T3SS effectors facilitate the maturation of the SCV, and can act as pro- or anti-inflammatory factors [22], [23].

Previously, our group found that while *S.* Enteritidis SPI-2 mutants were slower to colonize the spleens and livers of chickens, the levels of the mutant and wild-type were similar by day 4 post-challenge [24]. A major mode of transport for *Salmonella* to systemic sites like the liver and spleen is likely within macrophages [25]. There is a vast array of conflicting evidence in the literature about the importance of the SPI-2 T3SS to the survival of *S.* Typhimurium and *S.* Enteritidis within macrophages. In this study, we demonstrate that in activated HD11 chicken macrophage-like cells, the SPI-2 T3SS does not contribute to survival of *S.* Typhimurium and *S.* Enteritidis.

## Methods

### Cloning and production of *Salmonella* pathogenicity island 2 mutants

Construction of the SPI-2 mutants used in this study have been described previously [24].

### Bacterial strains and growth conditions

Bacterial strains used in this study are described in Table 1. Standard growth procedures were followed using Luria-Bertani (LB) broth and agar at 37°C.

**Table 1:** List of bacterial strains used in this study.

Bacterial strain	Short name	Properties	Source
*E. coli* DH5α	DH5α	F^-^, ϕ80dlacZΔM15, Δ(*lacZYA-argF*)U169, *deoR*, *recA1*, *endA1*, *hsdR17*(r_K_^−^, m_K_^+^), *phoA*, *supE44*, λ^−^, *thi-1*, *gyrA96*, *relA1*	Invitrogen
*S.* Typhimurium SL1344	SL1344	A wild-type *S.* Typhimurium strain, streptomycin resistant	Dr. B. Finlay^a^
*S.* Typhimurium SL1344 Δ*ssaR*	Δ*ssaR*	SL1344 missing the *ssaR* gene from SPI-2	Dr. B. Finlay^a^
*S.* Enteritidis Sal18	Sal18	Virulent for birds, invades liver and spleen, and colonizes gut	Dr. C. Poppe^b^
*S.* Enteritidis Sal18 ΔSPI-2::*cat*	ΔSPI-2::cat	Sal18 with the whole SPI-2 region deleted and replaced by a chloramphenicol resistance gene using the λRed system.	[24]
*S.* Enteritidis Sal18 ΔSPI-2	ΔSPI-2	Derivative of Sal18 ΔSPI-2::*cat* with the chloramphenicol resistance gene deleted using the λRed system.	[24]

a Dr. B. Finlay, University of British Columbia, Vancouver, British Colombia.

b Dr. C. Poppe, Laboratory for Foodborne Zoonoses, Health Canada, Guelph, Ontario.

### HD11 cell line and growth conditions

HD11 cells were kindly provided to VIDO by Dr. Kirk C. Klasing (currently Department of Animal Science, University of California – Davis, Davis, CA, USA). HD11 cells are a macrophage-like immortalized cell line derived from chicken bone marrow and transformed with the avian myelocytomatosis type MC29 virus [26]. HD11 cells were maintained at 42°C, in a humidified incubator (5% CO_2_), in RPMI 1640 media (Gibco) supplemented with 10% fetal bovine serum (FBS), 2 mM L-glutamine (Gibco), and 10 mM HEPES. For all assays involving bacteria the medium was changed to RPMI 1640 containing 10% heat-inactivated FBS, 2 mM L-glutamine, and 10 mM HEPES, before cells were seeded into 24-well cell-bind plates (Corning). HD11 cells were used for all assays between passages 15 and 25.

### Gentamicin protection assay

**Figure 1:**
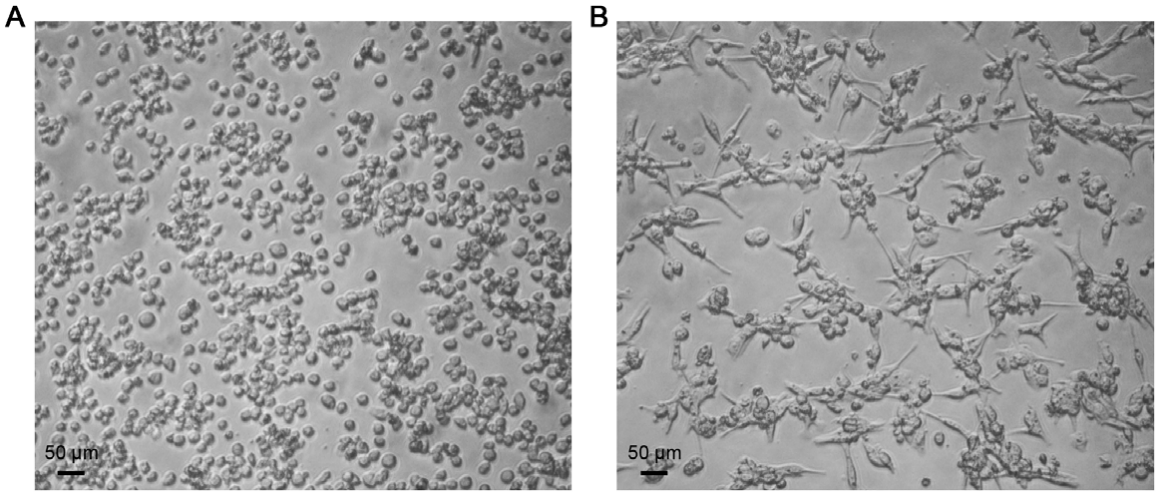
HD11 chicken macrophage-like cells. **Panel A** shows HD11 cells that have not been stimulated with PMA, while **Panel B** shows HD11 cells 12 hours after stimulation with PMA. Photographs were taken under 10X magnification.

Approximately 12 hours before infection, HD11 cells were placed in RPMI 1640 (10% heat-inactivated FBS, 2 mM L-glutamine and 10 mM HEPES) containing 100 ng/ml phorbol 12-myristate 13-acetate (PMA) (Sigma-Aldrich), and seeded into 24-well cell-bind plates (Corning) at a concentration of 5×10^5^ cells per well. At this time growth of bacterial strains were started. 12 hours post-activation, HD11 cells were checked to ensure that differentiation was induced by PMA (Figure 1) and bacterial overnight cultures were sub-cultured and grown to an OD_600_ corresponding to 1×10^8^ CFU/ml. The number of viable HD11 cells was determined from three wells via trypan blue exclusion in order to calculate the MOI. Media was removed from the cells, and bacteria (in pre-warmed RPMI 1640) were added to each well at a multiplicity of infection (MOI) of 25 (time 0 h). Serial dilutions of the bacteria were made and plated in order to confirm that each strain was added to the HD11 cells at an MOI of approximately 25. Plates containing HD11 cells and bacteria were subject to centrifugation at 200 x *g* in a Sorvall benchtop centrifuge for 5 minutes at room temperature, and then placed at 42°C. After 0.5 h, media containing bacteria was carefully removed from the HD11 cells, and the cells were washed once with PBS containing 500 µg/ml gentamicin. RPMI 1640 containing 250 µg/ml gentamicin was then added to each well, and the plates were placed back at 42°C. At each time point (0.5, 3, 6, 12 and 24 hours post-uptake [PU]), media was removed from three wells per bacterial strain and centrifuged at 20,800 x *g* in an Eppendorf benchtop microfuge for 10 minutes at 4°C. Sediments from this media fraction were resuspended in 0.5 ml 1% Triton X-100 (Sigma), and 100 µl portions from each sample were plated on LB-agar using 3 mm borosilicate glass beads. The cell monolayer from three wells, per bacterial strain, were washed once with PBS, and lysed in 0.5 ml 1% Triton X-100 in PBS. Dilution series of the cell monolayer fractions were made and 100 µl of each dilution (10^−4^, 10^−3^, 10^−2^, 10^−1^, 10^0^) was plated on LB agar using borosilicate glass beads. In order to confirm the effectiveness of the gentamicin, at the 0 h time point bacteria were also added to one 14 ml culture tube containing 5 ml of RPMI and one containing 5 ml of RPMI with 250 µg/ml gentamicin added. 100 µl from each tube was plated on LB agar at each time point. This experiment was repeated three times, and data pooled for statistical analysis.

### Location of *Salmonella* in the media fraction

It was important to determine if the bacteria in the media fraction were free, or contained within detached HD11 cells or cell fragments. A portion (100 µl) of the media fraction from HD11 cells infected with *S.* Typhimurium strain SL1344 from the 3 h PU time point was plated on LB agar to see if the bacteria will still viable in the media fraction, which contained gentamicin. The media fraction was then divided into two 5 ml portions in 14 ml culture tubes. To one tube, 1% Triton X-100 was added in order to lyse any eukaryotic cell membranes, and the tubes were placed in a shaker at 37°C for 3 hours, following which a further 100 µl from each tube was plated on LB agar. A portion of the initial media fraction was also subject to staining with Giemsa or PKH26 (Sigma-Aldrich) and viewed using a fluorescence microscope (Zeiss Axiovert 200M). PKH26 is a red fluorescent dye linked to long aliphatic tails that can insert into lipid regions of cell membranes. PKH26 and Giemsa staining were carried out as indicated by the manufacturer.

### Immunofluorescence

Survival assays were performed as above, but in 8 well chamber slides instead of 24-well plates, and bacterial numbers were not enumerated by plating. Instead, at each time point (0, 0.5, 3, 6, 12, and 24 hours PU), the media fraction was collected, sedimented, and resuspended in 100 µl 0.1% EDTA in PBS. The samples from the media fraction were then placed on slides using a CytoSpin 4 centrifuge (Thermo Scientific). Briefly, slides were placed in the CytoSpin centrifuge, and 100 µl of FBS was added and centrifuged onto the slides at 400 x *g* for 3 minutes, followed by the addition of 100 µl of sample under the same conditions. Slides were allowed to dry overnight, and fixed in ice-cold acetone for 10 minutes. After fixation, slides were washed 3 times with PBS, and then incubated for 0.5 h with fluorescein-conjugated mouse anti-*Salmonella* IgG or fluorescein-conjugated rabbit anti-*E. coli* IgG (1/50 in PBS). Samples were washed as before, and then treated with rhodamine-conjugated goat anti-mouse IgG or rhodamine-conjugated goat anti-rabbit IgG (1/50 in PBS). Samples were washed, and then treated with 0.5% Triton X-100 for 5 minutes to permeabilize HD11 cell membranes. Following an additional wash, goat anti-*Salmonella* IgG conjugated with fluorescein or rabbit anti-*E. coli* IgG conjugated with fluorescein (1/50 in PBS) were added to the samples. All antibodies were purchased from AbD Serotec. Samples were washed, and then stained with DAPI (10 µg/ml) (Sigma-Aldrich) for 15 minutes at room temperature. Coverslips were added to the slides using FluorSave™ mounting medium (EMD chemicals INC), and all samples were viewed using the Zeiss Axiovert 200M microscope with a mercury vapour short-arc lamp for fluorescence. Photographs were taken using the Zeiss Axiocam and were processed using Adobe© Photoshop© CS 5 for Mac OS X. Manipulations included cropping for space and level adjustment to reduce background noise.

### Superoxide assay

The assay to measure superoxide production via luminometry was adapted from Thrasher *et al.* [27]. Briefly, 12 hours before the experiment, 2×10^7^ HD11 cells were seeded into two 75 cm^2^ tissue culture flasks. PMA was added to one of the flasks at a concentration of 100 ng/ml. The next day cells were washed once with PBS and harvested by the addition of 0.5% trypsin. Cells were counted, and amounts corresponding to 5.0×10^5^ cells were added to individual eppendorf tubes prior to being centrifuged at 2700 x *g* for 5 min at 4°C, and resuspended in 100 µl Hanks' buffered saline (HBS) with calcium and magnesium (137 mM NaCl, 5.4 mM KCl, 358 mM NaHCO_3_, 0.44 mM KH_2_PO_4_, 0.34 mM Na_2_HPO_4_, 0.5 mM CaCl_2_, 1 mM MgCl_2_). Directly before reading luminescence, 100 µl of 10 µM luminol (Sigma-Aldrich) in HBS and 10 U of horseradish peroxidase (HRP) (Sigma-Aldrich) in 50 µl HBS were added to the cells. Samples were read for 5 seconds at 3–5 minute intervals using a GloMax 20/20 luminometer (Promega Biosciences).

### Statistical analysis

Data from each survival assay were pooled and ranked using Microsoft® Excel™ for Mac OS X. All statistical analyses were performed using GraphPad Prism® 5.0 for Mac OS X. One-way ANOVAs were performed on each time point. If significance between groups was found, the data was further analyzed using the post-hoc Tukey test. *p-*values≤0.05 were considered significant. Data from two superoxide assays were ranked and a two-way ANOVA was performed, followed by the post-hoc Bonferroni multiple comparisons test.

## Results

### Activation of HD11 cells

**Figure 2:**
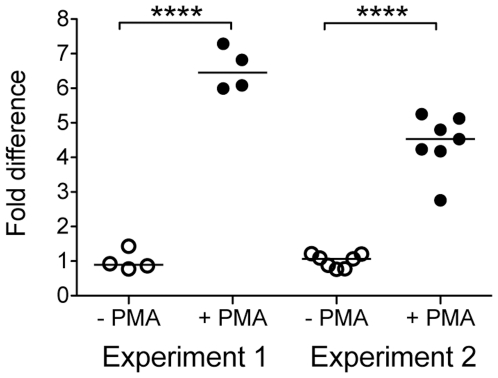
Hydrogen peroxide production by activated HD11 cells. Fold difference in hydrogen peroxide production in HD11 cells 12 hours after stimulation with PMA compared to that of unstimulated cells, as measured by luminescence produced by the reaction between hydrogen peroxide, luminal, and HRP.

HD11 cells are normally loosely adherent to plastic and have an ovoid shape; but can be activated to become more macrophage-like after stimulation with PMA (Figure 1, panel A). Twelve hours after exposure to 100 ng/ml PMA, the HD11 cells become more adherent to plastic, and their morphology becomes further macrophage-like, with a spindle shape (Figure 1, panel B). In addition, stimulated cells were found to produce more reactive oxygen species than unstimulated cells, as measured by production of hydrogen peroxide (*p*-value<0.0001) (Figure 2).

### Survival of *Salmonella* pathogenicity island 2 mutants within activated HD11 cells

**Figure 3:**
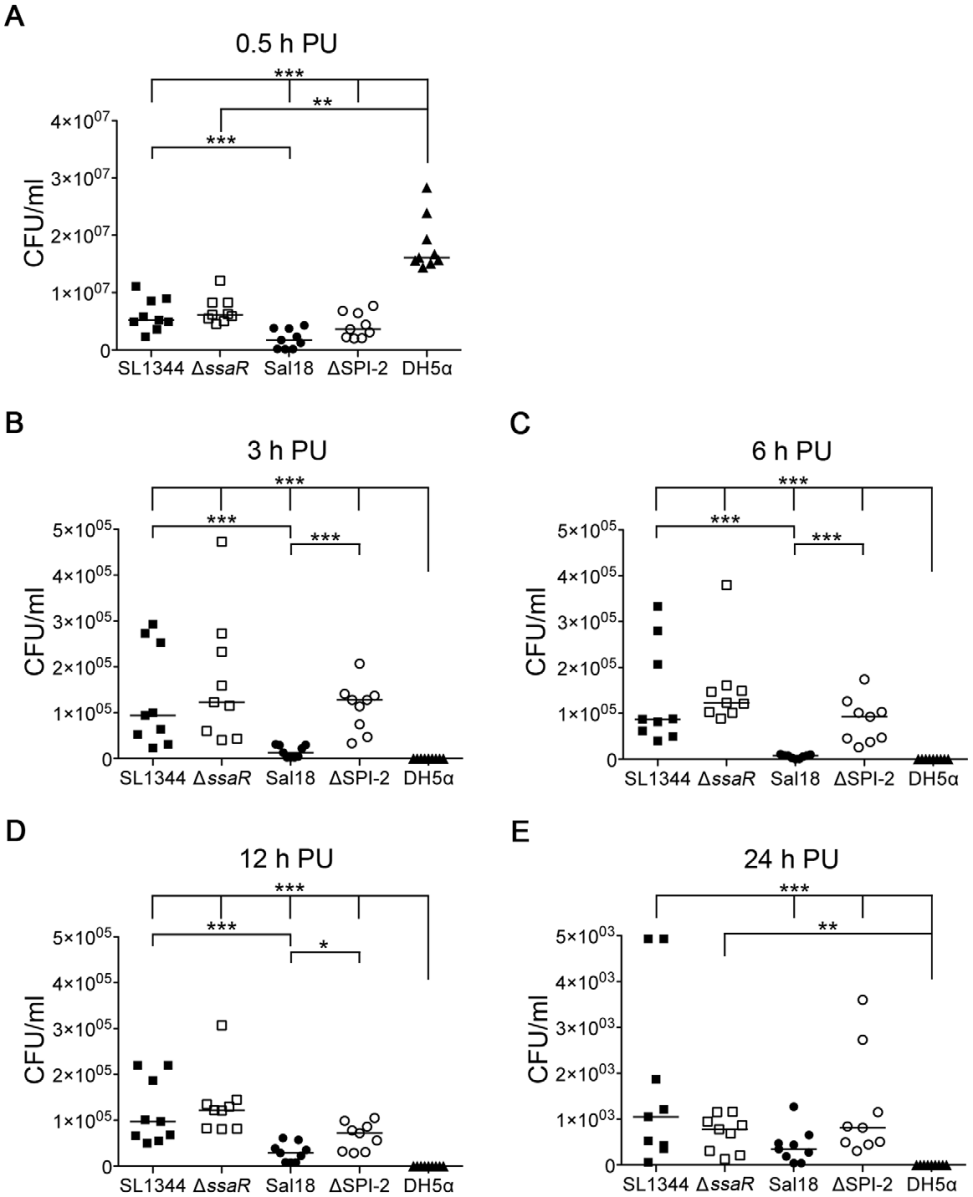
Recovery of *Salmonella* from the cell monolayer fraction of HD11 cells over time. The ability of SPI-2 mutants (*S. *Typhimurium Δ*ssaR* and *S.* Enteritidis ΔSPI-2) to survive in the chicken macrophage HD11 cell line was compared to their parent wild-type strains (*S.* Typhimurium SL1344 and *S.* Enteritidis Sal18) as well as to the non-pathogenic *E. coli* strain DH5α. **Panel A** shows the recovered CFU/ml from the cell monolayer fraction at 0.5 h post-uptake (PU), prior to addition of gentamicin. **Panels B**, **C**, **D**, and **E** show the recovered CFU/ml from the cell monolayer fraction after the addition of gentamicin at 3, 6, 12, and 24 h PU respectively. *, *p-*value<0.05; **, *p*-value<0.01; ***, *p*value<0.001. Note that the scale of the Y-axis is linear, and differs between time points.

At 0.5 hours PU, the non-pathogenic *E. coli* DH5α strain was recovered from the cell monolayer fraction at much higher levels than were any of the *Salmonella* strains (SL1344, Sal18, ΔSPI-2 *p*-value<0.001, and Δ*ssaR p*-value<0.01) (Figure 3, panel A). At 3, 6, 12, and 24 h PU, viable *E. coli* DH5α were no longer recoverable from the cell monolayer fraction (all *p-*value<0.001, except Δ*ssaR* at 24 h PU: *p*-value<0.01) (Figure 3, panels B–E). This observation indicates that even though more viable *E. coli* DH5α bacteria were recovered 0.5 h PU compared to the *Salmonella* strains, it is clear that the HD11 cells are able to effectively kill the *E. coli* strain after the initial infection process. Non-viable (or non-recoverable) *E. coli* DH5α are still visible by immunofluorescence at all time points, although in less abundant amounts than the *Salmonella* strains (data not shown). When the *Salmonella* strains were examined it was found that more of the *S.* Typhimurium wild-type strain (SL1344) was recovered from the cell monolayer fraction than the wild-type *S.* Enteritidis strain (Sal18) at all time points, with statistical significance observed at 0.5, 3, 6 and 12 h PU (*p-*value<0.001) (Figure 3). There was no difference in recovery between the wild-type and SPI-2 mutant (Δ*ssaR*) *S.* Typhimurium strains, while greater amounts of the *S.* Enteritidis SPI-2 (ΔSPI-2) mutant were recovered than the wild-type *S.* Enteritidis strain at 3, 6 (*p*-values<0.001), and 12 h PU (*p*-value<0.05) (Figure 3).

### Bacteria in the media fraction

**Figure 4:**
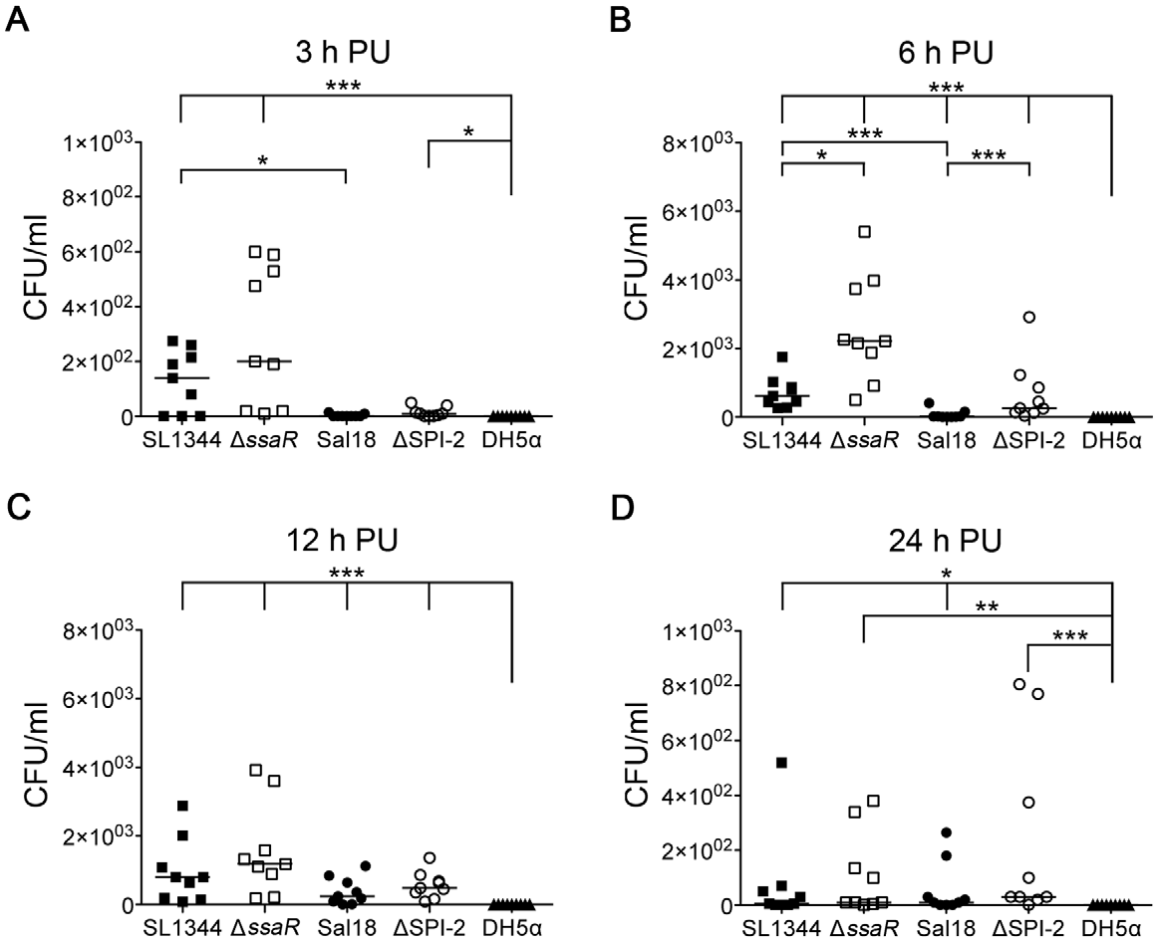
Recovery of *Salmonella* from the media fraction of HD11 cells over time. The ability of SPI-2 mutants (*S. *Typhimurium Δ*ssaR* and *S.* Enteritidis ΔSPI-2) to survive in the chicken macrophage-like HD11 cell line was compared to their parent wild-type strains (*S.* Typhimurium SL1344 and *S.* Enteritidis Sal18) as well as to the non-pathogenic lab *E. coli* strain DH5α. Surprisingly, *Salmonella*, but not *E. coli*, was recovered from the media at all time points PU following the addition of gentamicin. **Panel A** shows the CFU/ml recovered from the media fraction at 3 h post-uptake (PU) while **Panels B**, **C**, and **D** show the CFU/ml recovered from the media fraction at 6, 12, and 24 h PU respectively. *, *p-*value<0.05; **, *p*-value<0.01; ***, *p*-value<0.001. Note that the scale of the Y-axis is linear, and differs between time points.

**Figure 5:**
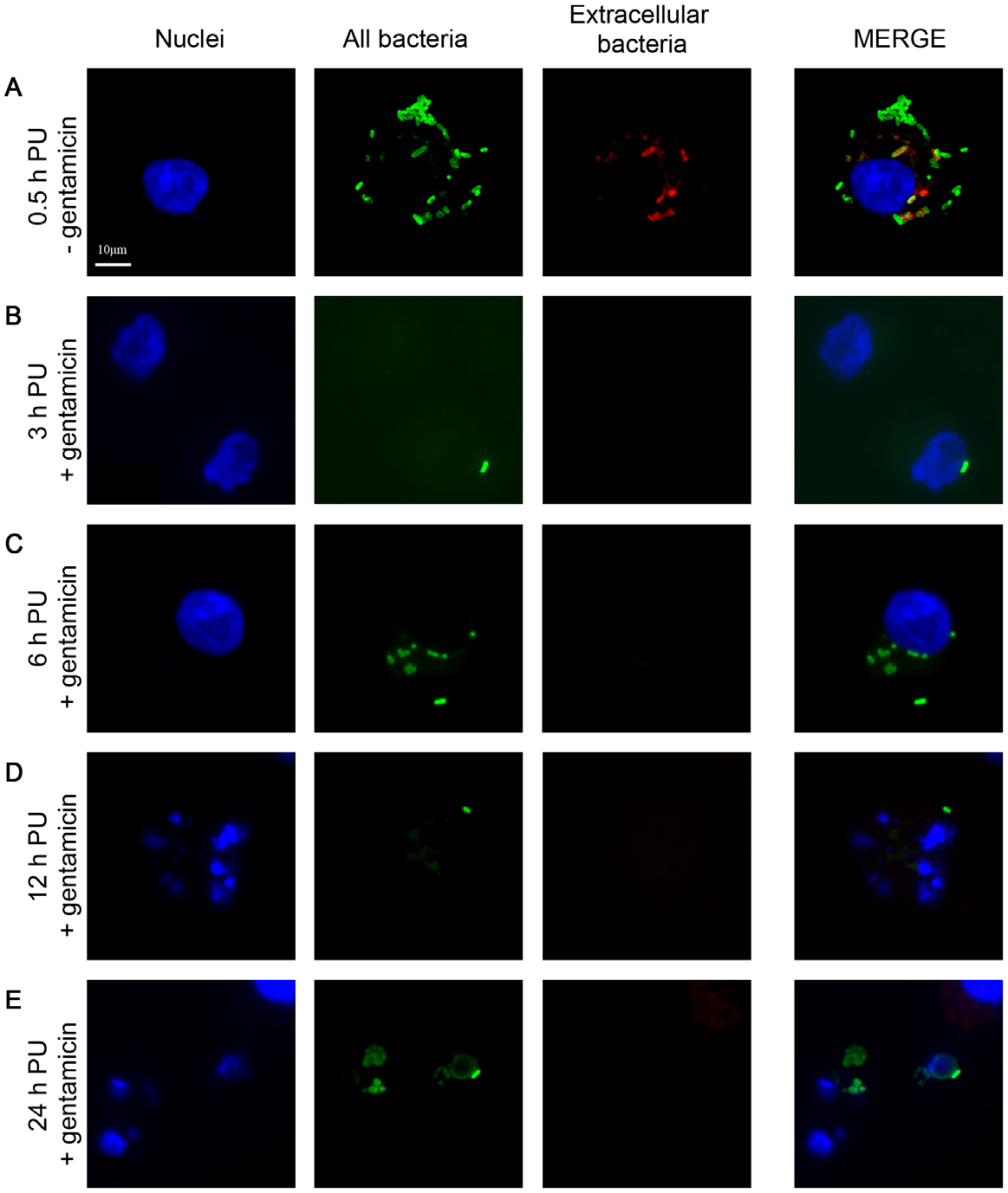
Wild-type *S.* Typhimurium strain SL1344 in the media fraction over time. HD11 cell nuclei are stained blue, both intra- and extracellular bacteria are green, and extracellular bacteria appear red or yellow. **Panel A** shows an HD11 cell loaded with wild-type *S.* Typhimurium strain SL1344 at 0.5 h PU, just prior to addition of gentamicin. **Panels B** and **C** show whole HD11 cells containing SL1344 at 3 and 6 h PU, respectively, after addition of gentamicin. **Panels D** and **E** show fragmented HD11 cells containing SL1344 at 12 and 24 h PU, respectively, after the addition of gentamicin. Whole and fragmented cells containing SL1344 were visible at all time points in the media fraction.

At 3, 6, 12, and 24 h PU (after the addition of gentamicin), *Salmonella* could be recovered from the media taken off the cell monolayer. When portions of the media fraction were added to either a tube containing RPMI and a tube containing RPMI with 1% Triton X-100, and grown for 3 hours, bacteria was only recoverable from the media fraction to which no Triton X-100 had been added. As 1% Triton X-100 is capable of disrupting eukaryotic, but not bacterial, cell membranes, this indicates that the bacteria are contained within eukaryotic cell membranes. Furthermore, PKH and Giemsa staining of the media fraction showed both whole cell and smaller membrane fragments in the media fraction (data not shown). At 3 and 6 h PU, more of the wild-type *S.* Typhimurium strain SL1344 was recovered from the media fraction than the wild-type *S.* Enteritidis strain Sal18 (*p-*values<0.05 and <0.001 respectively) (Figure 4, panels A and B). At 6 h PU, both mutant strains (Δ*ssaR* and ΔSPI-2) were recovered in greater amounts than their respective parent strains (SL1344 and Sal18) (Figure 4, panel B). Immunofluorescence at 0.5, 3, 6, 12, and 24 h PU showed that most *Salmonella* were associated with whole HD11 cells that have detached from the monolayer (Figure 5, panels A, B, and C), or fragmented cells (Figure 5, panels D and E). *Salmonella* was found to be sensitive to gentamicin, except when in the media fraction, until the addition of 1% Triton X-100. This, along with the aforementioned microscopy, indicates that the bacteria found in the media fraction are contained within whole and fragmented cells, where they are protected from the action of gentamicin. Finally, *E. coli* strain DH5α was not recoverable from the media fraction at any time point (Figure 4).

## Discussion

Chicken macrophage-like HD11 cells can be induced to be more macrophage-like by stimulation with PMA, as evidenced by morphology and production of reactive oxygen species (Figures 1 and 2, respectively). At 0.5 h PU, prior to addition of gentamicin, more *E. coli* DH5α were recovered from the cell monolayer fraction than the *Salmonella* strains. It is unclear why this may be, as similar numbers of intracellular and extracellular bacteria were present in all samples when visualized by immunofluorescence microscopy (data not shown). However, this microscopy does not differentiate viable from killed bacteria and it is possible that more live *E. coli* DH5α bacteria are initially phagocytosed by the macrophages, or that more *E. coli* DH5α remain associated with (but not phagocytosed) by the cells after the washing process. Following the addition of gentamicin, activated HD11 cells effectively killed *E. coli* DH5α, as viable *E. coli* DH5α were not recoverable from either the cell monolayer fraction or media fraction at any time point past 0.5 h. In comparison, all *Salmonella* strains were recoverable up to 24 h PU from HD11 cells. At most time points, the *S.* Typhimurium strain appeared to survive better within the HD11 cells than the *S.* Enteritidis strain. Importantly, at no time did the wild-type *S.* Typhimurium or *S.* Enteritidis strains survive better than their respective SPI-2 mutant strains, and, at 3, 6, and 12 h PU, the *S.* Enteritidis SPI-2 mutant (ΔSPI-2) out-performed the wild-type strain. All strains, including non-recoverable *E. coli* DH5α, were visible within macrophages at all time points PU by immunofluorescence, but it is likely that the immunofluorescence was detecting killed but intact, as well as viable, bacteria (data not shown). Typically only one or two bacteria were seen within an individual cell, although a few instances of large bacterial load were also observed. After the addition of gentamicin, fewer macrophages containing *E. coli* were visible, when compared to those infected with *Salmonella* strains.

In a mouse model of infection, multiple groups have shown that various SPI-2 mutants are highly attenuated in virulence (measured by LD_50_) [6], [28], [29], [30]. Initially, Hensel *et al.* showed that *S.* Typhimurium SPI-2 mutants replicated at similar levels to wild-type *S.* Typhimurium in the mouse macrophage-like RAW264.7 cell line [31]. However, later work by the same group (and others) indicated that SPI-2 mutants failed to replicate as well in mouse macrophages than wild-type strains if they were first grown to stationary phase and then opsonized before infection to enhance uptake of bacteria by macrophages [6], [28], [29]. In our experiments, bacteria were grown to mid-log phase before infection, and were not opsonized, because these conditions do not mimic the initial stages of infection by *Salmonella*. Many groups have recently published results in accordance with our findings. Forest *et al*. [32] determined that the absence of a functional SPI-2 T3SS in serovar Typhi (*S.* Typhi) did not affect survival in human macrophages. Aussel *et al.* [33] demonstrated that *S.* Typhimurium containing a non-functional SPI-2 or SPI-1 T3SS was able to survive similarly to wild-type *S.* Typhimurium in both mouse bone marrow-derived macrophages and RAW264.7 macrophages, but that the same mutants did not replicate to similar levels of the wild-type strain *in vivo*. A study by Helaine *et al.* [34] determined that while SPI-2 is important for replication of *S.* Typhimurium in macrophages, it does not affect the survival of phagocytosed bacteria within the SCV. Furthermore, it was shown in the study by Helaine *et al*. that most wild-type *S.* Typhimurium that are taken up by macrophages do not undergo replication at all, but rather enter a dormant state within the SCV. The number of bacteria in stasis did not differ between wild-type macrophages, *phox* ^−*/*−^ macrophages, or macrophages stimulated with IFNγ. Thus, *Salmonella* may be able to survive within macrophages without replicating and disseminate to systemic sites, regardless of the presence of SPI-2. In fact, it has been shown that SPI-2 mutants are able to reach the livers and spleens of mice and that similar numbers of spleen cells are infected, but mice infected with SPI-2 mutants have a reduced overall bacterial load in these organs [30], [34]. Previous work by our group showed that although *S.* Enteritidis SPI-1 and SPI-2 mutants were recovered in the livers and spleens of infected chickens in less abundance than the wild-type *S.* Enteritidis strain initially, the mutant strains reached comparable levels to the wild-type strain by day 4 PC [24], [35].

It is well known that the production of reactive oxygen species (ROS) by phagocytes is important for control of intracellular pathogens. Humans, or animals, with mutations in NADPH oxidase (Phox) are prone to severe recurrent infections by fungi and intracellular bacteria, including *Salmonella* [21], [36]. Phox assembles on phagosomes that contain intracellular pathogens, and is responsible for the production of superoxide (O_2_^−^). Superoxide is not readily able to cross the membranes of bacteria, but can spontaneously dismutate, or be dismutated by superoxide dismutases, into hydrogen peroxide (H_2_O_2_). Hydrogen peroxide can easily diffuse across bacterial membranes and can form highly reactive hydroxyl (HO) radicals in the presence of iron (Fe^2+^) that damage bacterial DNA, proteins, and lipids [21], [33]. *Salmonella* has developed multiple defenses to this process. *Salmonella* has two periplasmic superoxide dismutases (SodCI and SodCII) that combat exogenous superoxide. It also expresses three known cytosolic catalases (KatG, KatE, and KatN) and three cytosolic peroxidases (SodA, SodB, and Tpx) that degrade hydrogen peroxide within the bacterial cytoplasm. SodCI and SodCII, along with the cytoplasmic peroxidase Tpx, have been shown to be important for survival of *S.* Typhimurium in mouse macrophages [33], [36], [37]. It has also been previously shown that SPI-2 is important for vesicular trafficking and the association of Phox with the SCV in human and mouse macrophages; this observation led researchers to propose that the SPI-2 T3SS was essential in avoiding the oxidative burst [36], [38], [39]. However, recent work by Aussel *et al.* [33] indicates that SodCI, SodCII, and Tpx are sufficient for *Salmonella* to overcome ROS, and that *S.* Typhimurium SPI-2 mutants perform similarly to wild-type *S.* Typhimurium *in vitro*. They found that, *in vivo*, the wild-type *S.* Typhimurium had increased replication in relation to the SPI-2 mutant in both wild-type and *phox* ^−*/*−^ mice (although both strains reached higher levels in the *phox* ^−*/*−^ mice), indicating that while SPI-2 is important for replication, it does not play a major role in evasion of ROS. While we showed that HD11 macrophages activated with PMA produced greater levels of hydrogen peroxide than non-activated macrophages (Figure 2), we did not see a major difference in survival of SPI-2 mutants compared to wild-type strains in these activated cells. This indicates that any avoidance of ROS in this case was independent of a functional SPI-2 T3SS. Slauch *et al.* [40] state that ROS in the SCV of infected cells may not be diminished by wild-type *Salmonella* as previously reported, so the role of SPI-2 in avoidance of ROS, as well as the importance of ROS in control of *Salmonella* infection, remains unclear.

SPI-2 has been shown to change cytokine and chemokine production by macrophages, including the HD11 cell line [16], [41], [42], [43]. In HD11 cells, PipB has been shown to be important in the down regulation of pro-inflammatory cytokines, indicating a role for SPI-2 in repression of the host's innate immune response [42]. SPI-2 has also been shown to be important in limiting the antigen presenting abilities of macrophages and dendritic cells to T cells [44], [45]. It may be that SPI-2 is less important for survival of *Salmonella* within macrophages, but more important in modulation of macrophage stimulation of other immune cells via cytokine/chemokine production and antigen presentation. This would better explain some of the differences seen between *in vitro* SPI-2 *Salmonella* mutant survival and *in vivo* SPI-2 *Salmonella* mutant vulnerability.

Surprisingly, in our study, *Salmonella* was recoverable in the media fraction at each time point after the addition of gentamicin. These bacteria were found to be associated with both whole detached cells and closed cell fragments. If the bacteria within the cell fragments are viable, this would be a novel way for *Salmonella* to avoid the host immune system between host cell death and uptake by other phagocytic cells.

Taken together, these results indicate that survival in activated chicken HD11 macrophage-like cells is likely SPI-2 independent. Further research into the role of the SPI-2 T3SS in systemic spread in chickens is needed. Specific experiments involving challenge of chickens with labeled wild-type and *Salmonella* SPI-2 mutants would allow real-time observation of how the strains disseminate throughout the chicken. This would also allow for the surveillance of the specific cell types that the two strains are residing in within the chickens. Further, after isolation of infected phagocytic cells from challenged chickens, specific cytokine and chemokine profiles of these cells could be undertaken to see how SPI-2 effects immune cell function, as well as study of ROS and RNS production. Rather than using the available immortalized cell lines, isolation of a primary chicken macrophage cell line is desirable. Observations on how the cytokine and chemokine profiles of these cells change over time, when infected with wild-type *Salmonella* compared to the SPI-2 mutant, as well as whether the lack of SPI-2 affects replication of the bacteria within these cells would be helpful. Characterization of how *Salmonella* induces cell death in primary chicken macrophages, and the role SPI-2 plays, would be very useful and further study of the media fraction in the gentamicin protection assay would be interesting to see if *Salmonella* is truly contained in small membrane vesicles that are able to be taken up by naïve macrophages, and continue the infection process.
